# Supplementary material for: UVC-Based Air Disinfection Systems for Rapid Inactivation of SARS-CoV-2 Present in the Air
Source: Pathogens. 2023 Mar 7;12(3):419. doi: 10.3390/pathogens12030419 (PMC10053150; doi:10.3390/pathogens12030419)
Supplement: Supplementary file 1 [file pathogens-12-00419-s001.zip › pathogens-1990264-supplementary.pdf]

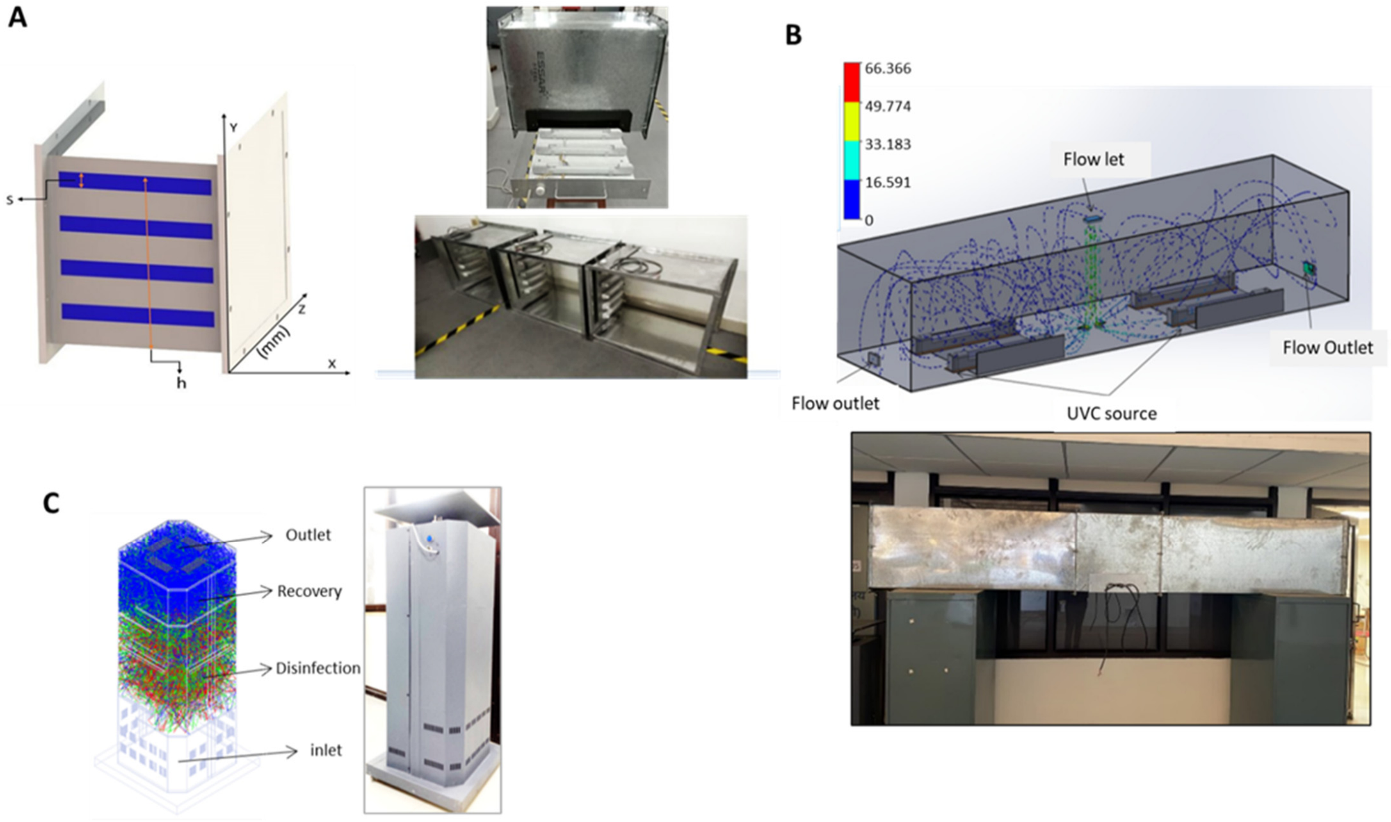

Supplementary Figure S1: Configuration of UVC disinfection systems. The figures are related to figure 1 and along with the CAD drawings actual photo of system is shown.
